# A NMDA Receptor Antagonist, MK-801 Impairs Consolidating Extinction of Auditory Conditioned Fear Responses in a Pavlovian Model

**DOI:** 10.1371/journal.pone.0007548

**Published:** 2009-10-26

**Authors:** Jun-Li Liu, Min Li, Xiao-Rong Dang, Zheng-Hong Wang, Zhi-Ren Rao, Sheng-Xi Wu, Yun-Qing Li, Wen Wang

**Affiliations:** 1 Department of Teaching and Training, Fourth Military Medical University, Xi'an, People's Republic of China; 2 Department of Psychology, School of Nurse, Third Military Medical University, Chong Qing, People's Republic of China; 3 Department of Nursing, Qing Hai Health and Professional Technology College, Xi Ning, People's Republic of China; 4 Department of Anatomy, Histology and embryology; K. K. Leung Brain Research Centre, Fourth Military Medical University, Xi'an, People's Republic of China; 5 Institute of Neuroscience, Fourth Military Medical University, Xi'an, People's Republic of China; University of Granada, Spain

## Abstract

**Background:**

In auditory fear conditioning, repeated presentation of the tone in the absence of shock leads to extinction of the acquired fear responses. The glutamate N-methyl-D-aspartate receptor (NMDAR) is thought to be involved in the extinction of the conditioned fear responses, but its detailed role in initiating and consolidating or maintaining the fear extinction memory is unclear. Here we investigated this issue by using a NMDAR antagonist, MK-801.

**Methods/Main Findings:**

The effects of immediate (beginning at 10 min after the conditioning) and delayed (beginning at 24 h after conditioning) extinctions were first compared with the finding that delayed extinction caused a better and long-lasting (still significant on the 20^th^ day after extinction) depression on the conditioned fear responses. In a second experiment, MK-801 was intraperitoneally (i.p.) injected at 40 min before, 4 h or 12 h after the delayed extinction, corresponding to critical time points for initiating, consolidating or maintaining the fear extinction memory. i.p. injection of MK-801 at either 40 min before or 4 h after delayed extinction resulted in an impairment of initiating and consolidating fear extinction memory, which caused a long lasting increased freezing score that was still significant on the 7th day after extinction, compared with extinction group. However, MK-801 administered at 12 h after the delayed extinction, when robust consolidation has been occurred and stabilized, did not affect the established extinction memory. Furthermore, the changed freezing behaviors was not due to an alteration in general anxiety levels, since MK-801 treatment had no effect on the percentage of open-arm time or open-arm entries in an Elevated Plus Maze (EPM) task.

**Conclusions/Significance:**

Our data suggested that the activation of NMDARs plays important role in initiation and consolidation but not maintenance of fear extinction memory. Together with the fact that NMDA receptor is very important for memory, our data added experimental evidence to the concept that the extinction of conditioned fear responses is a procedure of initiating and consolidating new memory other than simply “erasing” the fear memory.

## Introduction

Pairing of a previously neutral environmental stimulus (CS: conditioned stimulus, usually a tone), with an aversive outcome (US: unconditioned stimulus, usually an electric footshock) is termed as fear conditioning. Presentations of that CS alone later on elicit characteristic fear responses, including freezing [1], [2], [3]. Fear conditioning phenomenon is generally accepted as an explanation for anxiety disorders such as post-traumatic stress disorders (PTSD) [4]. Thus the extinction of conditioned fear is taken as one feasible mechanism for effective therapy including current extinction-based behavioral therapies [5].

Conditioned fear responses can be depressed by two approaches: facilitating fear extinction [6] or inhibiting fear reconsolidation [7], [8], [9]. It is commonly recognized that N-methyl-D-aspartate receptor (NMDAR) is required for the acquisition of fear conditioning, since intraamygdala infusions of NMDARs antagonists block the establishment of conditioned fear [10], [11], [12]. Interestingly, the NMDAR has also been implicated in both extinction and reconsolidation of conditioned fear [13]. Therefore, NMDAR blockade can maintain conditioned fear via impairing extinction or reduce conditioned fear via disrupting reconsolidation.

Although extensive studies have been conducted on fear extinction and the possible contribution of NMDAR to consolidating fear extinction memory [14], [15], [16], there are still some important but unclear points that need to be investigated. First, the long lasting effects of immediate or delayed extinction need to be confirmed. Several studies indicate that delayed extinction is better than immediate extinction [17], [18], but the possible long lasting (at least several days) effect of delayed extinction was not reported. Second, the detailed involvement of NMDAR in initiating, consolidating and maintaining extinction memory is not clear since most of the current studies focus on its contribution to initiation by using pre-extinction blockade [14], [16]. Third, in previous similar studies, the fear memory was reactivated by the CS (tone) that was intermingled with the shock context because the freezing behaviors were measured in the same shock chamber [14], [16]. It is not clear about the effect of NMDAR blockade on the “pure” CS conditioned fear responses. To answer this question, a predominant extinction should be established. The length and number of trials for the extinction session are key factors which determine the direction of fear reconsolidation or extinction during the “extinction” training [19], [20], [21]. When the session is brief, reconsolidation processes are dominant, whereas longer sessions induce predominant extinction [19], [20], [21].

Here, by using an extinction-predominant model induced by long extinction session [16], we first evaluated the long-lasting effect of immediate or delayed extinction. Then, we administered noncompetitive NMDAR antagonist (+) – 5 - methyl- 10, 11 – dihydro – S*H* - dibenzo [a,d] cyclohepten - 5, 10 - imine maleate (MK-801) intraperitoneally, at 40 min before, 4 h or 12 h after delayed extinction, to determine the effects of NMDAR blockade on initiating, consolidating or maintaining the fear extinction memory. General anxiety behaviors were evaluated with Elevated Plus Maze (EPM) to test whether the changed freezing behaviors were due to the effects of MK-801 on general anxiety rather than on fear extinction. Our study offered experimental evidence that delayed extinction has a long-lasting effect and MK-801, systemically administered pre- or post-extinction can impair the initiation and consolidation but not maintenances of fear extinction memory without affecting the animals' general anxiety behavioral pattern.

## Materials and Methods

### Subjects and drugs

Male Sprague–Dawley rats (8-week-old) were obtained from the animal center of the Fourth Military Medical University (Xi'an, PR China) and housed three to a cage, maintained on a 12-h light/dark cycle (light on from 08:00–20:00), and fed and watered ad libidum. All rats were habituated to the experimental room for 6 d before experiment. All animal work was approved by the Committee of Animal Care and Use for Research and Education at the Fourth Military Medical University. Each behavioral test was conducted during the light phase of the cycle (9:00 A.M.–5:00 P.M.) using independent experimental groups consisting of 7–16 animals per group. MK-801 (Sigma, St. Louis, MO; Catalog No.: M107; Batch No.: 027K4621; 5 mg) was stored in DMSO stock solutions (50 mM) and freshly diluted in 0.1 M PB (pH = 7.4). The solving vehicle was used as vehicle control for i.p. injections. According to the experiment plans, MK-801 solution was i.p. injected 40 min before, 4 or 12 h after extinction at the dose of 0.3 mg/kg. Such a relatively high dosage [14], [15], [16] was used to offer a complete blockade of NMDAR and it did not produce obvious side effects such as increased baseline freezing behaviors, increased general anxiety behaviors, etc in our pilot experiment **(Figure S1)** as well as in a previous report [22].

### Fear conditioning and extinction

Fear conditioning and extinction as well as retention of the extinction memory were performed in two different boxes: Box A that served for acclimation and fear conditioning and Box B that served for extinction training and retention tests. Box A was consisted of a modified shuttle box (W×D×H: 24.2×24.2×30 cm, Shanghai Mobiledatum Information Technology Co., Ltd, Shanghai, China) constructed of four vertical Plexiglas sides. Box B was a flat floored-cylinder consisted of vertical Plexiglas with a diameter of 24.2 and height of 30 cm. Box A had a floor made up of horizontal metal bars (0.5 cm diameter, spaced 1.5 cm apart) connected to an electric shock generator. Boxes A and B were placed inside a sound-attenuating chamber (Shanghai Mobiledatum Information Technology Co., Ltd, Shanghai, China) with a plastic floor. A speaker connected to a sound generator was also mounted at the top of the sound-attenuating chamber for the presentation of discrete tones. The experiments were carried out under dim light (4 lux) each day at almost the same time.

On day 6 upon arrival, rats were initially placed in Box A and left to explore the environment for 2 min. The tone habituation was done by presenting four tones (amplitude: 80 dB; frequency: 4 kHz, sine wave, totally 2 min) alone during which baseline freezing behaviors were recorded. The similar procedure was done in box B to measure baseline freezing behaviors and both protocols produced similar results.

On the following day (day 7), animals were placed in box A and left to explore the environment for 2 min followed with 10 tone-shock paired trainings. The conditioned stimulus was a tone (amplitude: 80 dB; frequency: 4 kHz, sine wave; duration: 30 s; inter-trial interval (ITI): 1–4 min) and the unconditioned stimulus was an electric shock (0.6 mA, 5 s, co-terminated with tone) delivered through the chamber floor bars.

Extinction trials were given in box B to animals 10 min (in experiment 1) or 24 h (in experiments 1 and 2) after the last conditioning trial. Retention tests were done in box B on day 9, 11,15 and 28 (refered as R 1, 3, 7 and 20 d (in experiment 1 only)). The extinction trials were 30 tones (ITI: 1–2 min; all other parameters were the same as for fear conditioning) in the absence of electric shock. For retention test, 5 tones (ITI: 1–2 min) were given. The chamber walls, floor, floor bars and tray underneath the floor were cleaned with 70% ethanol between each session.

Freezing during the presentation of CSs alone, defined as complete immobility of the animal in a stereotyped crouching position, except for movements necessary for breathing, was used as a memory index in the current study [23]. The position and shape of rats were dynamically returned based on computer-aided contrast-detecting image processing and calculation. Judged by the set threshold for immobility, the freezing times were calculated. All these image processing and calculation were accomplished with the Dr. Rat rodents' Behavior system (Shanghai Mobiledatum Information Technology Co., Ltd). Freezing behaviors during 5 randomly selected CSs were analyzed from the video recorded via a camera positioned above the operant chamber for 10-min observing period (5–9 CSs) after the last CS-US pairing in fear conditioning or during retention tests, by presenting only CS with a random ITI of 1–2 min. Freezing score is calculated as the percentage of freezing time to the total observing time (150 sec) ([TFreezing/Ttotal] ×100%). After the 10-min video recording, rats were returned to their home cages.

### EPM

EPM test was done according to our previous report [24]. The Plexiglas apparatus consisted of a plus-shaped platform elevated 50 cm from the floor. Two of the opposing arms (50 cm×10 cm) were enclosed by 40 cm-high side and end walls (closed arms, CA), whereas the other two arms had no walls (open arms, OA). Rats were placed individually into the center (neutral) zone of the maze, facing an OA and were allowed to explore the maze for a 5 min period. The number of open and closed arm entries and time spent in the open and closed arms were recorded. Animals were considered to be in the open or closed arms only when all four paws crossed out of the neutral zone. The EPM relies on the animal's natural fear of open spaces, and the percent of time spent in OA and percent of OA entries are believed to be measures of general anxiety level. The percentage of OA time was calculated by taking the time spent in the OA and dividing it by the sum of the time spent in the open and closed arms. The percentage of OA entries was calculated by taking the number of OA entries and dividing it by the sum of the entries into both open and closed arms.

### Experiment schedule

Rats were aclimated to the experimental room for 6 days with the baseline behaviors measured on the 6th d, and then trained to acquire the conditioned fear on day 7. Then they were used in experiment 1 or 2.

#### Experiment 1: Long-lasting effects of immediate or delayed extinctin

After acquiring conditioned fear responses on day 7, rats were randomly devided into the following 3 groups: 1. Conditioned fear group: drug free rats subjected to the fear conditioning and were kept for further behavioral tests (**Fear**), in these rats a gradual decrease of freezing behaviors can be observed (forgetting conditioned fear); 2. Immediate extinction group: rats subjected to the extinction trials 10 min after the last conditioned fear training (**Imme EXT**); 3. Delayed extinction group: rats subjected to the extinction trials 24 h after the last conditioned fear training (**EXT**). On day 9, 11, 15 and 28, half rats from each group were used for retention test in Box B and the other half were used for evaluating general anxiety behaviors with EPM. The two sets of rats received repeated tests in either Box B or EPM.

#### Experiment 2: effect of MK-801 on initiating, consolidating and maintaining extinction memory

We administered MK-801 at 4 h before, an early (4 h) or late (12 h) stage to investigate the effect of NMDAR blockade on the initiation, consolidation and maintenance of fear extinction memory. After acquiring conditioned fear on day 7, rats were randomly divided into the following 7 groups: 1. Conditioned fear group: drug free rats subjected to the fear conditioning and were kept for further behavioral tests (**Fear**); 2. Extinction group (equal to the delayed extinction group in experiment 1): rats subjected to the extinction trials 24 h after the conditioned fear training (**EXT**); 3. MK-801 pre-administration group: rats subjected to the delayed extinction trials and MK-801 was i.p. injected at the dose of 0.3 mg/kg at 40 min before the extinction (**MK-801 + EXT**); 4. Early stage MK-801 post-administration group: rats subjected to the delayed extinction and MK-801 was i.p. injected at the dose of 0.3 mg/kg at 4 h after the extinction (**EXT + MK-801 (4 h)**); 5. Late stage MK-801 post-administration group: rats subjected to the delayed extinction and MK-801 was i.p. injected at the dose of 0.3 mg/kg at 12 h after the extinction (**EXT + MK-801 (12 h)**); 6. Extinction group receiving vehicle treatment: rats subjected to the delayed extinction and 0.1 M PB (the volume was calculated according to the body weight) were injected at 4 h after the last extinction trial (**EXT + Veh**); 7. Extinction group receiving pre-administration of vehicle: Optimal volume of 0.1 M PB were injected and 40 min later, these rats were subjected to the delayed extinction (**Veh + EXT**). On day 9, 11, and 15, half rats from each group were used for retention tests in Box B and the other half were used for evaluating general anxiety behaviors with EPM.

Rats received vehicle injection at either 40 min pre- (**Veh + EXT**) or 4 h post- (**EXT + Veh**) extinction showed similar behavioral performance in either freezing behaviors or EPM test to the EXT rats. Naive rats received i.p. injection of MK-801 at the dose of 0.3 mg/kg or Veh injection had no change in baseline freezing score and general anxiety behaviors measured 40 min after the injection compared with the naive rats (**Figure S1)**.

### Statistical analysis

All data were analyzed using analysis of variance (ANOVA). Two-way ANOVA was used for analysis of freezing score and general anxiety behaviors (Treatments × Days). The LSD *Post hoc* test was used whenever appropriate and significance was accepted at 5% level (P<0.05).

## Results

### Baseline freezing score and general anxiety behaviors

**Figure 1:**
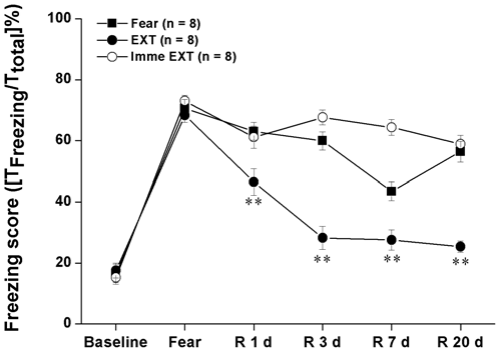
Experiment 1: Freezing score measured in rats receiving different extinction protocols. The freezing score were measured before the experiment (baseline), on the fear conditioning day (Fear) or during the retention tests (R 1, 3, 7 and 20 d). ** P<0.01, vs. Fear. Fear, conditioned fear rats underwent forgetting; EXT, conditioned fear rats underwent delayed extinction (beginning at 24 h post-conditioning); Imme EXT, conditioned fear rats underwent immediate extinction (beginning at 10 min post-conditioning).

The baseline freezing scores, measured in Box B, of Fear, Imme EXT, EXT, Ext + Veh, Veh + EXT, MK-801 + EXT, EXT + MK-801 (4 h) and EXT + MK-801 (12 h) groups were not significantly different (**Figs. 1** **and** **2**). There were no significant differences in the baseline general anxiety behaviors as entries into OA and percentages of time spent in OA of Fear, Imme EXT, EXT, Ext + Veh, Veh + EXT, MK-801 + EXT, EXT + MK-801 (4 h) and EXT + MK-801 (12 h) groups (**Figs. 3** **and** **4**).

**Figure 2:**
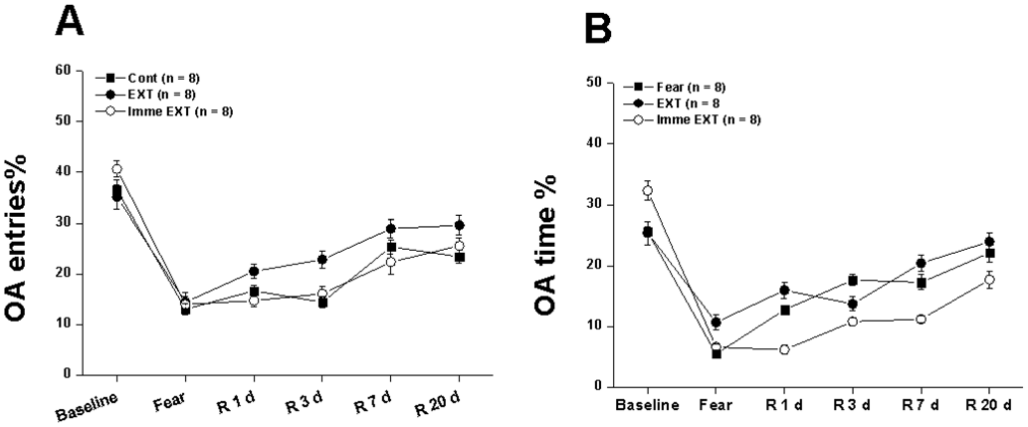
Experiment 2: Freezing score measured in rats receiving different treatments. The freezing score were measured before the experiment (baseline), on the fear conditioning day (Fear) or during the retention tests (R 1, 3 and 7 d). Fear, conditioned fear rats underwent forgetting; EXT, conditioned fear rats underwent delayed extinction (beginning at 24 h post-conditioning); MK-801 + EXT, rats subjected to the delayed extinction trials and MK-801 was i.p. injected at the dose of 0.3 mg/kg 40 min before the extinction; EXT + MK-801 (4 h), rats subjected to the delayed extinction and MK-801 was i.p. injected at the dose of 0.3 mg/kg 4 h after the extinction; EXT + MK-801 (12 h), rats subjected to the delayed extinction and MK-801 was i.p. injected at the dose of 0.3 mg/kg 12 h after the extinction; EXT + Veh, rats subjected to the delayed extinction and 0.1 M PB (the volume was calculated according to the body weight) were injected at 4 h after the last extinction trial; Veh + EXT, Optimal volume of 0.1 M PB were injected and 40 min later, these rats were subjected to the delayed extinction.

**Figure 3:**
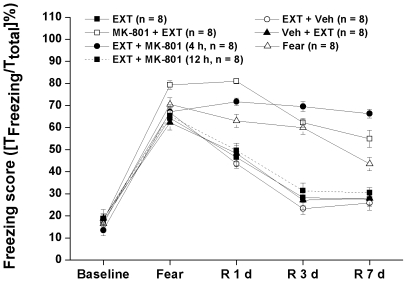
Experiment 1: General anxiety behaviors from different groups measured with EPM. The percentage of entries into OA (A) and time spent in OA (B) were calculated at different time points from rats that were naïve to the followed retention tests. Fear, conditioned fear rats underwent forgetting; EXT, conditioned fear rats underwent delayed extinction (beginning at 24 h post-conditioning); Imme EXT, conditioned fear rats underwent immediate extinction (beginning at 10 min post-conditioning).

**Figure 4:**
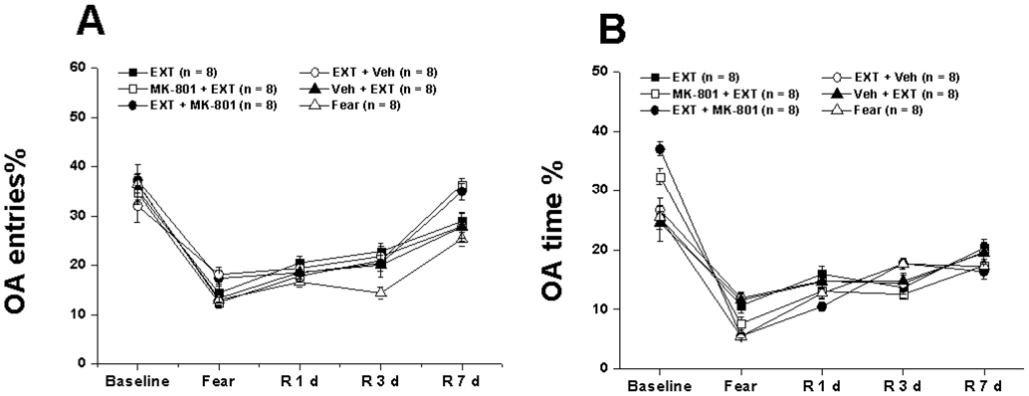
Experiment 2: General anxiety behaviors from different groups measured with EPM. The percentage of entries into OA (A) and time spent in OA (B) were calculated at different time points from rats that were naïve to the followed retention tests. EXT, conditioned fear rats underwent delayed extinction (beginning at 24 h post-conditioning); MK-801 + EXT, rats subjected to the delayed extinction trials and MK-801 was i.p. injected at the dose of 0.3 mg/kg 40 min before the extinction; EXT + MK-801 (4 h), rats subjected to the delayed extinction and MK-801 was i.p. injected at the dose of 0.3 mg/kg 4 h after the extinction; EXT + MK-801 (12 h), rats subjected to the delayed extinction and MK-801 was i.p. injected at the dose of 0.3 mg/kg 12 h after the extinction; EXT + Veh, rats subjected to the delayed extinction and 0.1 M PB (the volume was calculated according to the body weight) were injected at 4 h after the last extinction trial; Veh + EXT, Optimal volume of 0.1 M PB were injected and 40 min later, these rats were subjected to the delayed extinction.

### Experiment 1: Long-lasting effect of immediate or delayed extinction

Our data indicated that delayed extinction caused a better and long-lasting depression on conditioned fear responses than that caused by immediate extinction. Two way ANOVA showed significant effects of protocol [F(2, 144) = 14.760, p<0.01], day [F(5, 144) = 52.512, p<0.01] and interaction between protocol and day [F(8, 144) = 10.247, p<0.01] on the freezing score (**Fig. 1**).

According to previous studies [17], [18], delayed extinction has a better extinction effect than immediate extinction, but the authors did not extend their observations to the possible long-lasting effect. Here, our data indicated that delayed extinction produced a stable depression on conditioned fear responses indicated by the decreased freezing score until 20 days after the extinction. *Post hoc* test suggested that the delayed extinction significantly decreased the freezing behaviors (P<0.01, vs. Fear), but immediate extinction did not (P>0.05, vs. Fear). The freezing behaviors were significantly decreased by delayed extinction at the first retention test (R 1 d, P<0.01) and existed during the whole observing window (P<0.01, for R 3, 7 and 20 d). However, immediate extinction produced a time-dependent depression on conditioned fear responses which might be caused by the unpaired CSs when repeated measuring the freezing score, just like what happens in the fear group. That there were no difference in the temporal change of freezing scores between immediate extinction and fear group supports the inefficiency of immediate extinction on the conditioned fear response.

### Experiment 1: Immediate or delayed extinction did not alter the general anxiety behavioral patterns

It is possible that delayed extinction could alter general anxiety behaviors which may affect the freezing behaviors. To test this possibility, we also compared the anxiety levels of these groups before the whole experiment (baseline, B) right after fear condition (Fear, F) and the time-points corresponding to retention tests ( R 1, R 3, R 7 and R 20 d). These rats were naïve to retention tests to make the situation relatively simple.

Two way ANOVA showed significant effects of protocol [F(2, 144) = 3.655, p<0.05], day [F(5, 144) = 79.778, p<0.01] and interaction between protocol and day [F(8, 144) = 2.562, p<0.05] (Fig. 2A) on the percentage of entries into OA (**Fig. 3A**). Two way ANOVA also showed no significant effects of protocol [F(2, 144) = 2.523, p>0.05], but significant effects of day [F(5, 144) = 72.166, p<0.01] and interaction between protocol and day [F(8, 144) = 6.166, p<0.01] (Fig. 2A) on the percentage of time spent in OA (**Fig. 3B**). *Post hoc* test indicated that there is no difference between groups (P>0.05). Thus, the significant effects of interaction between protocol and day originated mainly from the source of day. Taking together, our data indicated that the general anxiety behaviors were decreased along with the experiment and the daily general anxiety behavioral patterns in Imme EXT and EXT groups were similar to those of Fear group (**Fig. 3**).

*Post hoc* test suggested that fear condition caused a significant increase of anxiety behaviors, and these anxiety behaviors gradually decreased (as indicated by the increased percentage of entries as well as time spent in OA of EPM) in all the testing groups. But the anxiety behaviors could not reach the baseline level measured before conditioned fear. It is possible that the adaptive tendency to the novel open space of OA can induce such a gradual increase in exploring OA of EPM (**Fig. 3**). To confirm this point, an experiment should be designed during which each rat is exposed to the EPM for only one time. Since the current design is already good enough for our purpose, the contribution of adaptive tendency to the gradual increase of exploring OA of EPM along with retention needs to be done in the future study.

### Experiment 2: MK-801 impaired initiation and consolidation but not retention of fear extinction memory

Our data indicated that MK-801 administered at either 40 min pre- or 4 h post-extinction impaired initiation and consolidation of fear extinction memory. However, once the extinction memory has been consolidated 12 h after the extinction trials, blocking NMDAR by MK-801 could not impair the retention of fear extinction memory. Two way ANOVA showed significant effects of treatment [F (6, 280) = 50.577, p<0.01], day [F (4, 280) = 39.889, p<0.01] and interaction between treatment and day [F(24, 280) = 50.787, p<0.01] on the freezing score (**Fig. 2**).

As showed in **Fig. 2**, forgetting can be observed in the Fear rats: without any extinction training, these rats can establish some extinction memory as indicated by the gradually decreased freezing score which was significant on R 3 d (P<0.05, vs. F). Delayed extinction protocol produced significant extinction of fear memory on R 1 d after the last extinction trial. This extinction memory reached peak on R 3 d and kept at a stable level until R 20 d (**Figs. 1** **and** **2**). Veh administered 40 min before or 4 h after the delayed extinction did not change the curve for extinction memory (**Fig. 2**). For EXT rats receiving MK-801 administration at 40 min before (MK-801 + EXT) or 4 h after (EXT + MK-801 (4 h)) the delayed extinction, the consolidation of fear extinction was significantly impaired on R 1 d (P<0.05 for both groups, vs. corresponding data of EXT group), furthermore, the freezing scores of these 2 groups on R 3 and 7 d were significantly higher than that of EXT group (P<0.05 on R 3 d; P<0.01 on R 7d; for both groups, vs. corresponding data of EXT group), indicating a long lasting impaired fear extinction memory. There was no significant difference between MK-801 + EXT or EXT + MK-801 (4 h) group and the Fear groups. Meanwhile, there was some slight difference between MK-801 + EXT and EXT + MK-801 (4 h) groups: rats receiving 4 h post-extinction treatments seem to keep some ability of consolidating fear extinction memory but without significant difference. MK-801 administered 12 h after the last extinction, when consolidation of fear extinction memory has occurred, did not change the freezing score in the followed retention tests (P>0.05, vs EXT, Veh + EXT or EXT + Veh, on each retention test day). All these findings suggested that manipulating NMDARs before the consolidation of fear extinction memory may offer some clinic clues for the therapy of anxiety disorders, which needs to be further investigated.

### Experiment 2: MK-801 treatment did not change the general anxiety behavioral patterns during extinction

It is possible that MK-801 altered general anxiety-like behaviors that were not involved in a condition component, producing long term effects on freezing behaviors. To rule out this possibility, we also tested whether MK-801 administration would alter anxiety levels before the whole experiment (Baseline) and on Fear, R 1, 3 and 7 d. These rats were naïve to freezing score measurements on Fear, R 1, 3 and 7 d to make the situation relatively simple.

Our data indicated that MK-801 administered at 40 min before, 4 h or 12 h after the delayed extinction did not change the general anxiety behavioral patterns that were observed in EXT, EXT+ Veh, or Veh + EXT groups (**Fig. 4**). Two way ANOVA showed no significant effects of treatment [F (6, 280) = 2.473, p>0.05] and interaction between treatment and day [F(12, 160) = 7.092, p>0.05] but significant effects of day [F(4, 280) = 148.320, p<0.01] (**Fig. 4A**) on the percentage of entries into OA (**Fig. 4A**).

Two way ANOVA also showed no significant effects of treatment [F(6, 280) = 2.087, p>0.05], but significant effects of day [F(4, 280) = 198.771, p<0.01] and interaction between treatment and day [F(24, 280) = 5.425, p<0.01] (**Fig. 4B**) on the percentage of time spent in OA (**Fig. 4B**). All data from the percentage of entries and time spent in OA collectively suggested a gradual decrease of anxiety behaviors (as increased exploration in OA of EPM) along with the observing window, and MK-801 treatment at either pre- or post-extinction (4 or 12 h ) did not change such a general anxiety behavioral pattern.

## Discussion

We first revealed that delayed extinction could produce a better and long-lasting depression on conditioned fear responses than immediate extinction. Using this protocol as a positive control, we investigated the contribution of NMDARs blocking to initiation, consolidation and maintenance of fear extinction memory. Systemic administration of MK-801 at 40 min before or 4 h after a long extinction session that caused the predominant extinction over reactivation of conditioned fear, caused impairment in initiation and consolidation of fear extinction memory. Meanwhile, MK-801, injected after the consolidation become robust at 12 h after the delayed extinction, did not affect the established fear extinction memory.

Our study first extends previous reports that delayed extinction can cause a “durable” effect which is still significant at R 2 d. We seek to confirm that the delayed protocol is better than immediate extinction and the effect is still significant at R 20 d. Extinction is generally accepted as an established memory of CS + noUS which competes with CS + US memory thus depress the conditioned fear responses, but not an erase of the established CS + US memory [19]. In a previous study conducted by Myers and coworkers [25], it was suggested that extinction conducted immediately after fear learning may erase or prevent the consolidation of the fear memory trace. Since extinction is a major component of nearly all behavioral therapies for human fear disorders, this finding supports the notion that therapeutic intervention beginning very soon after a traumatic event will be more efficacious. But this report was challenged by two more recent reports in which immediate extinction is inferior to delayed extinction for depressing conditioned fear responses in both human beings [17] and animals[17], [18]. However, the long-term effect of delayed extinction was not reported. Our data offered evidence, that delayed extinction can induce a long-lasting depression of conditioned fear responses. Our report, together with these two previous reports [17], [18], supported that a delayed intervention might be more efficacious than immediate one.

The role of NMDARs blockade with either systemic or focal injection (to amygdala) of MK-801 has been widely studied [14], [15], [26], [27], which offered evidence that MK-801 can impair the reactivation as well as extinction of conditioned fear. It is suggested that re-exposure to CS can cause either reactivation (a single CS presentation) or extinction (CS re-exposed many times) of conditioned fear [19]. Thus, the effect of NMDARs blockade on conditioned fear depends critically on the parameters of re-exposure to the CS during memory reactivation or extinction training [19]. Our purpose is to investigate the contribution of NMDARs blockade to the initiating, consolidating and maintaining fear extinction memory, thus, a long extinction session that included 30 repeated unpaired CS presentations was employed in the current study to produce predominant extinction over reactivation of conditioned fear responses [16]. Our data showed that blockade of NMDARs, before extinction memory was initiated and consolidated, impaired the consolidation of fear extinction, which is consistent with previous studies [28] and may suggest that some partial NMDARs agonists (e.g. D-cycloserine) could help get a good therapeutic effect on an anxiety patient during the intensive intervention period. On the other hand, after the extinction memory was established at 12 h after the delayed extinction, blockade of NMDARs did not affect the freezing scores measured in retention tests later on. Thus, our data collectively suggested that NMDARs are involved in initiating and consolidating but not maintaining fear extinction memory. Consistent with other studies using different modalities of learning and memory, the ionotropic glutamate receptors (including AMPA, NMDA and KA types) change on the membranes of synaptic site (majorly post-synaptic) is believed to contribute to establishing short-term learning [28], [29] (also personal communication with R Shigemoto and Y Fukazawa in National Institute for Physiological Sciences of Japan), while some structural changes including density, length and shape of synapses, are suggested to be involved in keeping the established memory (Our unpublished data by using a motor learning paradigm, also personal communications with Dr. R Shigemoto). Furthermore, such structural changes were suggested to be NMDAR (majorly NR2B) dependent [30]. As for the extinction memory, future morphological studies need to be conducted to offer direct evidence.

Although similar to previous studies, there are still some discrepancies that need to be addressed.

We measured the freezing behavior to CS in a novel chamber to reflect the real “fear response to CS”. One commonly used protocol to measure the freezing behaviors is to put the animals back to the shock chamber after conditioned fear and then the unpaired CS were given to induce freezing behaviors [14], [16]. Under such circumstance, the freezing behavior is believed to be induced by both CS and the contextual cue of the chamber where the traumatic events (feet shock) occurred during the conditioning. Thus, the freezing behaviors are complicated [3]. To make our model and measurement simple and specific to the CS, we measured the freezing behaviors to CS in a novel chamber without introducing the extra traumatic contextual cues. Thus, the explanation of our data might be more straightforward than others in which the CS and contextual cues induced freezing behaviors together [14], [16].

In summary, in the current study we found that delayed extinction protocol produced better and long-lasting depressive effect on the conditioned fear responses than immediate extinction. Using an extinction predominant model, before establishing robust and stable fear extinction memory, systemic administration of MK-801 impaired the initiation and consolidation of fear extinction memory without altering the general anxiety behavioral patterns. However systematic administration of MK-801 after the robust fear extinction memory has been established, did not affect the established memory. Together with the fact that NMDARs is very important for memory, our data added experimental evidence to the concept that the extinction of conditioned fear responses is a procedure of initiating and consolidating new memory other than simply “erasing” the fear memory.

## Supporting Information

Figure S1 MK-801 as well as vehicle injectiondid not affect the general anxiety behaviors as well as freezing behaviors measured 40 min later, as compared with naïve rats. Naïve, rats without any treatment upon arrival; Naïve + MK-801, MK-801 was i.p. injected at 40 min before behavioral measurements at the dose of 0.3 mg/kg; Naïve + Veh, Optimal volume of vehicle was i.p. injected at 40 min before behavioral measurements at the dose of 0.3 mg/kg.
(2.07 MB TIF)
